# Medical student perceptions of working in clinical environments during the COVID-19 pandemic

**DOI:** 10.7189/jogh.10.020380

**Published:** 2020-12

**Authors:** Jay J Park

**Affiliations:** Edinburgh Medical School, University of Edinburgh, Edinburgh, UK

The coronavirus disease 2019 (COVID-19) pandemic has caused major disruption to medical education for student doctors in the United Kingdom (UK). This has led to widespread cancellations and postponement of clinical placements, electives and examinations.

On March 24, 2020, the UK Government announced that 5500 final-year medical students would have the option of joining the National Health Service (NHS) workforce early to offer additional support and help relieve the increasing strain put on clinical services by the spread of COVID-19 [1]. This was on a voluntary basis [2]; however, a number of concerns had been raised regarding appropriate supervision, induction, access to Personal Protective Equipment (PPE), insurance, mental well-being and continued education [3].

**Figure:**
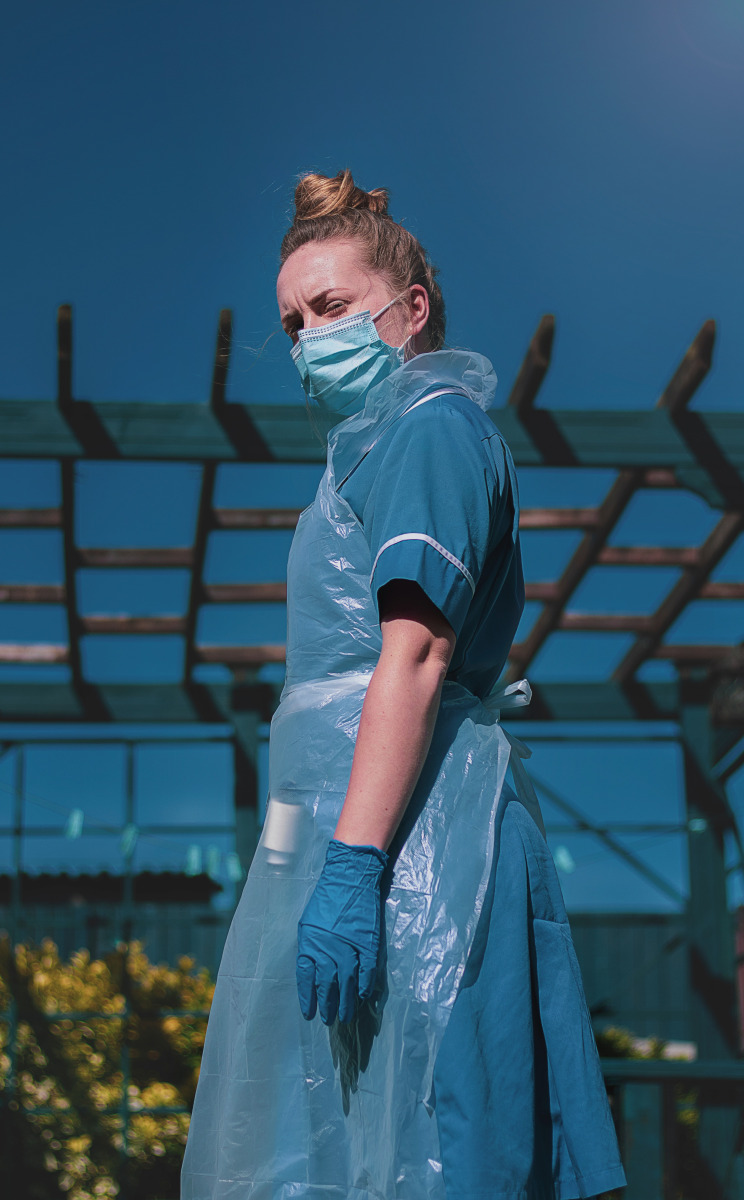
Photo: By Luke Jones, from unsplash.com.

We sought to determine the thoughts of UK medical students regarding their willingness to be placed in clinical environments during the COVID-19 outbreak. Medical students across the UK were invited in the period between March 13 and March 26, 2020, via social media, to participate in an online survey; 206 students completed the survey (Appendix S1 of the **Online Supplementary Document**). 26.7% (n = 55) of the respondents were final year medics, 49.5% (n = 102) were in Year 5, and 13.1% (n = 27) were in Year 4 and 10.7% (n = 22) were in Years 1-3.

62.6% (n = 129) of students said they would be willing to attend clinical placements during the COVID-19 outbreak. 58.1% (n = 75) of this group said they were willing to be on the wards despite feeling it would be an unsafe environment for them.

With regards to mitigating risks for medical students, 69% (n = 144) felt that their medical school had taken adequate measures to reduce their exposure to COVID-19. The remaining 31.1% (n = 64) highlighted frustrations surrounding a delayed response in offering specific advice regarding working in clinical environments and reducing the risk of exposure to the virus. Multiple responses demanded clearer guidelines from their medical schools and instructions for the appropriate use ofPPE. The majority (80.6%, n = 166) agreed there have been measures put in place by the medical school to reduce their risk of exposure to the virus (**Figure 1**). Furthermore, 35.9% (n = 74) of respondents said they were still willing to attend placements despite being worried about being infected in a clinical setting. From the clinical years, 43.6% (n = 24) of the final year students, 30.4% (n = 31) of year 5 students, and 51.9% (n = 14) of year 4 students said they will attend placement despite feeling unsafe. Also, 46.6% (n = 96) has stated that their attendance will not be affected by the COVID-19 outbreak.

**Figure 1:**
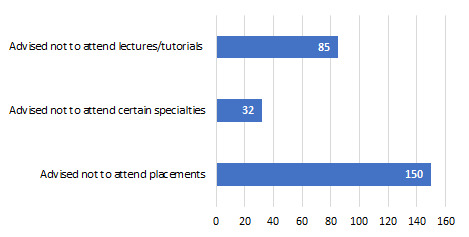
COVID-19 measures enforced by medical schools in March 2020.

This survey suggests that despite concerns surrounding safety and risk of exposure to COVID-19, most UK medical students were willing to work in clinical environments. This may have translated into a significant number of final-year medical students taking up the opportunity to support the NHS during the pandemic.

## Additional material

Online Supplementary Document

## References

[1] Schraer R. ExCeL Centre to be used as coronavirus hospital. BBC News. 2020. Available: https://www.bbc.com/news/health-52018477. Accessed: 25 September 2020.

[2] MSC issues Statement of Expectation for medical student volunteers in the NHS | Medical Schools Council. Available: https://www.medschools.ac.uk/news/msc-issues-statement-of-expectation-for-medical-student-volunteers-in-the-nhs. Accessed: 25 September 2020.

[3] Ahmed H Allaf M Elghazaly H. COVID-19 and medical education. Lancet Infect Dis. 2020;20:777-8. 10.1016/S1473-3099(20)30226-7 32213335 PMC7270510

