# Supplementary material for: Medical student perceptions of working in clinical environments during the COVID-19 pandemic
Source: J Glob Health. 2020 Nov 23;10(2):020380. doi: 10.7189/jogh.10.020380 (PMC7688067; doi:10.7189/jogh.10.020380)
Supplement: Online Supplementary Document [file jogh-10-020380-s001.pdf]

COVID-19 Medical Student (Clinical attachment Years) Perception and Impact Survey

Corona Virus Disease 2019 (COVID-19) was recently classified as a pandemic and cases have been increasing in the UK. We are interested in finding out the impact this has had on medical student in their clinical years. (Clinical years strictly refers to those on placements with patient contact in a clinical setting) Please take 2 minutes to fill in this survey.

If you'd like to get in touch with us you can contact our team at s1512134@sms.ed.ac.uk

*Required fields

1. Which medical school at you attending? *

- University of Aberdeen School of Medicine and Dentistry
- Anglia Ruskin University School of Medicine
- Aston University Medical School
- Barts and The London School of Medicine and Dentistry
- University of Birmingham College of Medical and Dental Sciences
- Brighton and Sussex Medical School
- University of Bristol Medical School
- University of Buckingham Medical School
- University of Cambridge School of Clinical Medicine
- Cardiff University School of Medicine
- University of Dundee School of Medicine
- Edge Hill University Medical School
- The University of Edinburgh Medical School
- University of Exeter Medical School
- University of Glasgow School of Medicine
- Hull York Medical School
- Imperial College London Faculty of Medicine
- Keele University School of Medicine
- Kent and Medway Medical School
- King's College London GKT School of Medical Education
- Lancaster University Medical School
- University of Leeds School of Medicine
- University of Leicester Medical School
- Lincoln Medical School
- University of Liverpool School of Medicine
- University of Manchester Medical School
- Newcastle University School of Medical Education
- Norwich Medical School
- University of Nottingham School of Medicine
- University of Oxford Medical Sciences Division
- Plymouth University Peninsula Schools of Medicine and Dentistry
- Queen's University Belfast School of Medicine

- University of Sheffield Medical School
- University of Southampton School of Medicine
- University of St Andrews School of Medicine
- St George's, University of London
- University of Sunderland School of Medicine
- Swansea University Medical School
- University of Central Lancashire School of Medicine
- University College London Medical School
- University of Warwick Medical School
- Other: please specify
- Prefer not to say

2. If other, please specify:

____

3. What is your current year of study? *

- Year 1
- Year 2
- Intercalated year
- Year 3
- Year 4
- Final year (5/6)
- Other: _______________________________

4. Has your medical school confirmed that your clinical placement has been or will be cancelled due to COVID-19? *

- Yes
- No

5. What placement are you currently attached to? *

____

6. Are you aware of the COVID-19 pandemic? *

- Yes
- No

7. Have you been asked to come in for hospital/GP placement at this time? *

- Yes
- No

8. How worried are you about being infected with COVID-19 during your current clinical placement? *

- Worried
- Unsure
- Not worried

9. Have you been given advice by your medical school regarding how to reduce your risk of exposure to COVID-19 while in clinical environments? *

- Yes
- No

10. If yes, please specify:

- ☐ Hand hygiene advice
- ☐ Use of masks
- ☐ Travel advice
- ☐ Avoid patients with suspicious symptoms
- ☐ Avoid potential aerosal generating procedures (NIV, tracheostomy, high flow nasal oxygen)
- ☐ Other: ______________________

11. Have you been given advice from your medical school regarding when you should be suspicious of a patient/yourself being infected with COVID-19? *

- Yes
- No

12. Have there been any measures put in place by your medical school to help you reduce your risk/exposure to COVID-19? *

- Yes
- No

13. If yes, please specify:

- ☐ Advised not to attend placements
- ☐ Advised not to attend certain specialties
- ☐ Advised not to attend lecture/tutorial settings
- ☐ Other: ______________________

14. Do you think your medical school/hospital has done enough to reduce your risk of COVID-19? *

- ◯ Yes
- ◯ No

15. If not, do you have any suggestions on what else they could do?

____

____

____

____

____

16. How safe do you feel in your clinical environment considering the current COVID-19 outbreak? *

- ◯ Safe
- ◯ Not safe but still willing to attend placement
- ◯ Not safe and reluctant to attend placement
- ◯ Other ______________________

17. Has your attendance for your clinical placement been affected by the COVID-19 outbreak? *

- Yes
- No

18. If yes, how often have you been attending your placement during the COVID-19 outbreak?

- Twice as usual
- More frequently than usual
- Less frequently than usual
- Half of usual
- Rarely
- Not at all
